# The role of rs2237781 within *GRM8* in eating behavior

**DOI:** 10.1002/brb3.151

**Published:** 2013-06-23

**Authors:** Marie-Therese Gast, Anke Tönjes, Maria Keller, Annette Horstmann, Nanette Steinle, Markus Scholz, Ines Müller, Arno Villringer, Michael Stumvoll, Peter Kovacs, Yvonne Böttcher

**Affiliations:** 1 Department of Medicine, University of LeipzigLeipzig, Germany; 2 IFB Adiposity Diseases, University of LeipzigLeipzig, Germany; 3 Department for Neurology, Max-Planck-Institute for Human Cognitive and Brain SciencesLeipzig, Germany; 4 Division of Endocrinology, Diabetes and Nutrition, University of Maryland School of MedicineBaltimore, Maryland; 5 Diabetes and Endocrinology Section, Veterans Administration Medical CenterBaltimore, Maryland; 6 Institute for Medical Informatics, Statistics and Epidemiology, University of LeipzigLeipzig, Germany; 7 LIFE Research Center, University of LeipzigLeipzig, Germany; 8 Clinic of Cognitive Neurology, University of LeipzigLeipzig, Germany

**Keywords:** Addiction, alcohol intake, food intake, human eating behavior, smoking behavior

## Abstract

**Introduction:**The *glutamate receptor*, *metabotropic 8* gene (*GRM8*) encodes a G-protein-coupled glutamate receptor and has been associated with smoking behavior and liability to alcoholism implying a role in addiction vulnerability. Data from animal studies suggest that *GRM8* may be involved in the regulation of the neuropeptide Y and melanocortin pathways and might influence food intake and metabolism. This study aimed to investigate the effects of the genetic variant rs2237781 within *GRM8* on human eating behavior. **Methods:**The initial analysis included 548 Sorbs from Germany who have been extensively phenotyped for metabolic traits and who completed the German version of the three-factor eating questionnaire. In addition, we analyzed two independent sample sets comprising 293 subjects from another German cohort and 430 Old Order Amish individuals. Genetic associations with restraint, disinhibition, and hunger were assessed in an additive linear regression model. **Results:**Among the Sorbs the major G allele of rs2237781 was significantly associated with increased restraint scores in eating behavior (*P* = 1.9 × 10^−4^; *β* = +1.936). The German cohort and the Old Order Amish population revealed a trend in the same direction for restraint (*P* = 0.242; *β* = +0.874; *P* = 0.908; *β* = +0.096; respectively). A meta-analysis resulted in a combined *P* = 3.1 × 10^−3^ (*Z*-score 2.948). **Conclusion:**Our data suggest that rs2237781 within *GRM8* may influence human eating behavior factors probably via pathways involved in addictive behavior.

## Introduction

Eating behavior has been shown to be a complex trait influenced by genetic and psychological factors as well as social and environmental circumstances influencing individual food selection, taste preferences, eating pattern, and eating behavior (Steinle et al. 2002; Grimm and Steinle 2011). A genetic contribution to individual eating behavior phenotypes has been demonstrated by heritability estimates (0.28, 0.40, and 0.23 for restraint, disinhibition, and hunger, respectively) in the Old Order Amish population, a genetically isolated Caucasian population of Central European dissent (Steinle et al. 2002). Numerous candidate gene studies support the role of genetics in eating behavior. For instance, genetic variation in *TAS2R38* has been significantly associated with eating behavior disinhibition in Old Order Amish (Dotson et al. 2010a) and genetic variation in bitter taste receptors has been reported to influence glucose homeostasis (Dotson et al. 2008, 2010b). Taste perception is predominantly mediated via G-protein-coupled receptors. The glutamate receptor 8 (GRM8) is a G-protein-coupled glutamate receptor influencing the inhibition of the cyclic AMP cascade as well as regulating the presynaptic glutamate release. Genetic variation within *GRM8* has been reported to significantly influence risk for diseases affecting the central nervous system including depression (Terracciano et al. 2010), autism (Li et al. 2008), schizophrenia (Takaki et al. 2004), and attention deficit hyperactivity syndrome (Elia et al. 2011). Interestingly, electrophysiological studies linked variants within *GRM8* to increased risk of vulnerability to alcoholism (Rangaswamy and Porjesz 2008; Chen et al. 2009). Furthermore, rs2237781 within *GMR8* has been identified to be at risk for smoking initiation and suggests that members of the glutamate receptor family may associate with nicotine dependence and vulnerability to addiction (Vink et al. 2009). The neurotransmitter glutamate is involved in substance abuse behavior and may influence food intake (Stanley et al. 1993). A glutamate injection into the lateral hypothalamus has led to a dose-dependent eating response in satiated rats (Stanley et al. 1993). Although the hypothesis of “food addiction” is under debate, there are further indications implying that alterations in brain reward pathways are similar to those seen in drug addiction, particularly through effects on the dopaminergic system (Johnson and Kenny 2010; Pandit et al. 2012). Several studies have shown that mechanisms influencing craving for alcohol and other substances may possibly overlap with processes regulating appetite for food, implying a potential relationship with eating behavior (Robinson and Berridge 2000; Kelley et al. 2005; Volkow and Wise 2005; Volkow et al. 2008, 2011, 2013). Moreover, there are indeed similarities reported for both eating disorders and substance abuse (Umberg et al. 2012). In line with this, data from studies in chicks indicate that *GRM8* may influence the NPY system and melanocortin pathway which may play a role in feeding behavior and metabolism via the hypothalamic pathway (Higgins et al. 2010). Taken together, *GRM8* might be involved in the control of addiction behavior and may play a role in the regulation of eating behavior phenotypes.

In the present study we aimed to assess the effects of the genetic variant rs2237781 within *GRM8* on eating behavior determined by the German version of the three factor eating questionnaire (TFEQ) (Pudel and Westenhöfer 1989) in the self-contained population of Sorbs (Veeramah et al. 2011), and to replicate the findings in two independent study cohorts.

## Methods

### Subjects

#### Sorbs

All subjects of the discovery cohort are part of an extensively phenotyped self-contained population in Eastern Germany, the Sorbs (Böttcher et al. 2009; Veeramah et al. 2011). The phenotyping included a standardized interview for past medical history, family history and eating behavior factors (German version of TFEQ, Pudel and Westenhöfer 1989), collection of anthropometric data (weight, height, waist-to-hip ratio, body impedance analysis), and a 75 g oral glucose tolerance test (OGTT). Moreover, data regarding alcohol intake (glasses per week, 0.2 L), smoking behavior (cigarettes per day), and coffee consumption (cups per day) have been recorded. In total, 618 Sorbs out of 1046 completed the German version of the TFEQ. Seventy subjects with Type 2 diabetes (T2D) have been excluded from the study (definition of T2D according to ADA criteria [ADA 2010]). Finally, the study included 548 Sorbs (346 females; 202 males). Mean age was 45 ± 16 years and mean body mass index (BMI) 26.1 ± 4.3 kg/m². Mean eating behavior scores for the Sorbs population are shown in Table 1.

**Table 1:** Mean eating behavior scores for the Sorbs, German cohort, and Amish cohort categorized by body mass index (BMI)

Eating behavior factor	Sorbs				German cohort				Amish cohort			
		
	BMI <25 (*n* = 235)	≤25 BMI <30 (*n* = 226)	BMI ≥30 (*n* = 84)	*P*-value	BMI <25 (*n* = 159)	≤25 BMI <30 (*n* = 48)	BMI ≥30 (*n* = 128)	*P*-value	BMI <25 (*n* = 145)	≤25 BMI <30 (*n* = 187)	BMI ≥30 (*n* = 157)	*P*-value
Restraint	7.4 ± 5.0	8.0 ± 4.6	9.2 ± 5.1	0.014	5.2 ± 4.5	7.3 ± 4.2	6.7 ± 4.5	0.002	5.4 ± 3.3	6.8 ± 4.2	8.6 ± 4.5	0.0001
Disinhibition	3.9 ± 2.6	4.5 ± 3.0	5.2 ± 3.8	0.002	4.7 ± 2.6	6.2 ± 2.8	7.4 ± 3.4	0.0001	4.1 ± 2.1	4.8 ± 2.3	6.9 ± 3.5	0.0001
Hunger	4.0 ± 2.8	3.7 ± 2.7	4.3 ± 3.3	0.219	5.3 ± 3.1	5.3 ± 3.0	5.7 ± 3.5	0.549	4.3 ± 2.6	3.9 ± 2.6	5.3 ± 3.3	0.0001

Sorbs, BMI missing for two individuals; Hunger score missing for one individual. *P*-values have been calculated by one-way analysis of variance (ANOVA).

The study was approved by the ethics committee of the University of Leipzig and all subjects gave written informed consent before taking part in the study.

#### German cohort

For replication purposes, we analyzed another sample set from Germany comprising 293 healthy volunteers (100 female, 193 male). Subjects were recruited in Leipzig, Germany, via newspaper announcements, posters in public transportation, and announcements on a local internet-based platform (overweight and obese subjects) or the local participant database of the Max Planck Institute for Human and Cognitive Brain Sciences. Phenotyping of participants included the following measurements: anthropometric data (BMI, weight, height), age, sex, smoking behavior, eating behavior factors were assessed by the German version of TFEQ (Pudel and Westenhöfer 1989). Mean age was 27 ± 5 years and mean BMI 27.7 ± 6.8 kg/m². Mean eating behavior scores for the German cohort are shown in Table 1. The local ethics committee of the University of Leipzig approved the study.

#### Old Order Amish (AFDS)

The Amish Family Diabetes Study (AFDS) is an effort to identify genetic contributors to obesity, diabetes, and cardiovascular diseases. Recruitment and phenotyping of AFDS participants has previously been described (Hsueh et al. 2000). Briefly, individuals with a previous diagnosis of T2D having an age at diagnosis between 35 and 65 years were recruited, as well as all first and second-degree family members over the age of 18 years of each proband (siblings, parents, offspring, grandparents, grandchildren, aunts, and uncles). Phenotyping included anthropometric measures (height, weight, waist circumference, hip circumference, and body impedance analysis), a 75 g OGTT (performed only among individuals not known to have a diagnosis of T2D), blood pressure, blood chemistry, and lipid profiles. Whole blood was collected for DNA extraction. To examine the relationship of eating behavior, obesity and related traits, and genetics, we administered the TFEQ (Stunkard and Messick 1985). For the purposes of the current analysis, we analyzed eating behavior scores from 430 individuals (417 nondiabetic subjects [96%]) from the AFDS (48% male). The mean age of men was 52.1 ± 11.8 years and of women 51.3 ± 11.7 years. Mean BMI was 26.5 ± 3.9 kg/m^2^ for men and 28.8 ± 5.5 kg/m^2^ for women. Eating behavior scores (restraint 6.9 ± 4.0; disinhibition 5.3 ± 2.6; hunger 4.5 ± 2.8) were positively associated with BMI (Steinle et al. 2002).

### German version of TFEQ

We investigated specific factors influencing eating behavior as evaluated by the German version of the TFEQ quantifying three different eating behavior factors influencing human eating behavior: dietary restraint, disinhibition, and hunger (Pudel and Westenhöfer 1989). We assessed all questions by assigning each item either with a score from 1 to 4 or true–false questions with 1 or 0. The restraint scale includes 21 questions measuring individual cognitive control of eating. The eating behavior factor disinhibition represents susceptibility to loose cognitive control by external factors resulting in overeating (16 questions). Realizing hunger feelings based on physiological signals leading to food intake was covered by 14 questions.

### Genotyping

*GRM8* variant rs2237781 was genotyped using the TaqMan SNP Genotyping assay (Applied Biosystems, Inc., Foster City, CA). The genotyping reaction was amplified on an ABI 2720 Thermal Cycler (Applied Biosystems Inc.; 95°C for 10 min, and 92°C for 15 sec, and 60°C for 1 min, for 40 cycles) and fluorescence was detected on an ABI 7500 Real-Time PCR System (Applied Biosystems Inc.). To assess genotyping reproducibility, a random ∼5% selection of the samples was re-genotyped in all SNPs; all genotypes matched initial designated genotypes. For replication of the association signals in the Old Order Amish, we used rs10487466 which serves as linkage disequilibrium proxy for rs2237781 (*r*^2^ = 1.0 based on HapMap release 27). The genotypes were extracted from a previously completed genome-wide scan using GeneChip Human Mapping 100K Set (Affymetrix, Santa Clara, CA) platform (Rampersaud et al. 2007). There were no deviations from Hardy–Weinberg equilibrium.

### Statistical analysis

Prior to statistical analysis, non-normally distributed parameters were logarithmically transformed to approximate normal distribution. Genetic associations with restraint, disinhibition and hunger were assessed using linear regression models (data analyzed as continuous variables) using age, gender, BMI, and current smoking as covariates for the Sorbs and German cohort. Significant effects in the Sorbs were adjusted for relatedness structure estimated on the basis of genome-wide SNP array data. The Amish data were adjusted for age, sex, and relatedness structure. Current smoking was defined as follows: current smokers versus ever smokers + never smokers. *P*-values <0.05 were considered to provide nominal evidence for association. Two-sided *P-*values are reported. Statistical analyses were performed using SPSS statistics 20 version 20.0.1 (SPSS, Inc., Chicago, IL). For adjustments regarding relatedness structure in the discovery cohort, we used a mixed-model approach implemented in the GenABEL package of the statistical software environment R (http://www.r-project.org, Amin et al. 2007). A meta-analysis was performed using METAL (http://www.sph.umich.edu/csg/abecasis/metal/). Study specific *P*-values and effect directions were converted into a *Z*-statistics and weighted with sample size of each study.

## Results

### Association analysis in Sorbs

An association analysis for variant rs2237781 located in intron 4 of *GRM8* was performed in the discovery population. Using linear regression analysis and an additive inheritance model the major G allele was significantly associated with higher restraint in eating behavior (adjusted *P* = 1.9 × 10^−4^) in the Sorbs (Table 2). No significant association could be detected for the eating behavior factors disinhibition and susceptibility to hunger feelings. Given a low frequency of the A allele (minor allele frequency [MAF 0.07]) we also included a recessive model of inheritance which showed significantly higher restraint values in homozygous G allele carriers (Table 2).

**Table 2:** Association analysis for rs2237781 with eating behavior factors under linear regression analyses

	Sorbs					German cohort					Amish cohort				
			
	GG	GA+AA	*P*-value	*β*	SE	GG	GA+AA	*P*-value	*β*	SE	TT	TC+CC	*P*-value	*β*	SE
Male/female	180/286	22/60				168/93	25/7				202/259	22/8			
Age (years)	45 ± 15.8	47 ± 14.1				27 ± 5.6	25 ± 4.4				53.0 ± 12.4	57.0 ± 11.6			
BMI (kg/m^2^)	26.0 ± 4.3	26.1 ± 4.6	0.766	+0.005	0.017	27.8 ± 7.6	28.4 ± 7.6	0.479	−0.023	0.032	28.0 ± 5.0	27.9 ± 4.0	0.729	−0.368	1.017
WHR	0.85 ± 0.1	0.85 ± 0.9	0.573	−0.008	0.014	n.a.	n.a.	n.a.	n.a.	n.a.	0.87 ± 0.07	0.90 ± 0.06	0.466	0.016	0.021
Restraint	8.1 ± 4.8	6.4 ± 4.7	0.00019^1^	+1.936^1^	0.515^1^	6.0 ± 4.6	5.1 ± 4.0	0.242	+0.874	0.746	7.0 ± 4.3	6.9 ± 3.9	0.908	−0.09	0.829
			0.00026^2^	+1.964^2^	0.534^2^										
Disinhibition	4.3 ± 3.0	4.3 ± 2.5	0.740	+0.104	0.313	5.9 ± 3.1	5.1 ± 3.4	0.222	0.616	0.503	5.2 ± 2.9	5.6 ± 2.7	0.284	0.683	0.636
Hunger	3.9 ± 2.9	3.9 ± 2.5	0.963	+0.015	0.326	5.5 ± 3.2	4.9 ± 3.0	0.615	0.286	0.568	4.4 ± 2.8	5.8 ± 3.6	0.004	1.797	0.620
Alcohol^3^	2.8 ± 1.1	2.6 ± 1.2	0.778	+0.034	0.120	n.a.	n.a.	n.a.	n.a.	n.a.	n.a.	n.a.	n.a.	n.a.	n.a.
Smoking^4^	10.1 ± 8.7	8.9 ± 6.0	0.767	+0.456	1.539	n.a.	n.a.	n.a.	n.a.	n.a.	n.a.	n.a.	n.a.	n.a.	n.a.
Coffee consumption^5^	1.8 ± 0.7	1.7 ± 0.6	0.420	+0.456	0.100	n.a.	n.a.	n.a.	n.a.	n.a.	n.a.	n.a.	n.a.	n.a.	n.a.

Data are presented as mean ± SD. *P*-values for eating behavior factors were calculated in a linear regression model with adjustment for age, gender, ln (BMI) (except for BMI), current smoking (except for smoking), and family structure (for Sorbs). *P*-values for consumer goods are adjusted for age and sex. SE, standard error; BMI, body mass index; WHR, waist-to-hip ratio.

1 Additive model of inheritance.

2 Due to small sample size of homozygous minor allele carriers we applied recessive model of inheritance (G/G vs. G/A+A/A and T/T vs. C/T+C/C for rs2237781 and rs10487466, respectively). n.a. not available; *β* indicates effect size and direction for the risk allele (major alleles G or T).

3 Number of glasses per week (general size 0.2 L).

4 Number of cigarettes per day.

5 Number of cups per day.

When analyzing data regarding alcohol intake, smoking behavior, and coffee consumption, we detected a higher but nonsignificant intake for each category in homozygous G allele carriers (Table 2).

### Association analysis in German cohort

In our second cohort comprising a limited sample set of 293 individuals we observed no significant association (Table 2). However, we detected the same effect direction between the major G allele as the allele show tendency for higher restraint values (Table 2). No significant association was detected for the eating behavior factors disinhibition and susceptibility to hunger feelings.

### Association analysis in Old Order Amish

Despite consistent effect directions with the discovery and the German cohort, no significant association was found between rs10487466 and restraint eating behavior in the Amish using linear regression models (adjusted *P* = 0.908, *β* = +0.096; Table 3). Of note, there was a significant association of rs10487466 with hunger (*P* = 3.9 × 10^−3^, adjusted for age, sex, and family structure, data not shown).

**Table 3:** Meta-analysis for association of rs2237781 with restraint including Sorbs, German cohort, and Old Order Amish

Chr	Effect allele	Sorbs			German cohort			Old Order Amish			Combined		
			
		*P*-value	*β*	SE	*P*-value	*β*	SE	*P*-value	*β*	SE	*P*-value	*Z*-Score	Direction
7	G	0.00018	+1.951	0.518	0.372	+0.685	0.765	0.908	+0.096	0.829	0.00319	2.948	+++

*P*-values were calculated based on effect sizes from linear regression model using additive inheritance model. All presented data are adjusted for age, gender, and family structure (for Sorbs, Amish). SE, standard error.

Meta-analysis is based on effect sizes from linear regression models.

### Meta-analysis including all three study populations

A sample size weighted meta-analysis including the results from all three study populations (Sorbs, German cohort, and Old Order Amish) resulted in a significant association for restraint (combined *P* = 3.1 × 10^−3^, *Z*-score 2.948, Table 3).

## Discussion

The metabotropic receptor GRM8 has been associated with smoking behavior (Vink et al. 2009) and liability to alcoholism (Chen et al. 2009) implying there may be a role in addiction vulnerability. Moreover, *GRM8* has been identified to be differentially regulated during different nutritional states in chicks (Higgins et al. 2010). It has been shown to be coregulated in the same gene network with *proopiomelanocortin* gene (*POMC*), one of the most important genes controlling metabolism, highlighting its potential role in food intake (Higgins et al. 2010). POMC is a central player of the melanocortin system within the arcuate nucleus of the hypothalamus (reviewed in Cone 2006). Higgins et al. (2010) described a reduced *POMC* expression in fasted chicks. The coregulation of *GRM8* and *POMC* suggests that glutamatergic neurotransmission may influence feeding behavior in chicks (Higgins et al. 2010). Glutamate itself is involved in addiction and may also influence food intake (Stanley et al. 1993). Furthermore, it has been postulated that common hedonic mechanisms may underlie obesity and drug addiction (Johnson and Kenny 2010). Filbey et al. (2012) reported further indications for a potential overlap of neural mechanisms in addiction and compulsive overeating adding further weight on possibly common regulatory processes.

In the present study we therefore hypothesized that rs2237781 within *GRM8* might influence human eating behavior factors in a similar fashion as seen in smoking behavior. Using linear regression models we observed in the Sorbs significantly increased restraint scores in individuals for the homozygous major G allele for rs2237781. Restraint eating is known to be a behavioral trait cognitively controlling body weight not only in normal weight individuals but also in obese and overweight subjects. Individuals representing restraint eating behavior tend to rigorously control, for example, the amount of food intake as well as caloric intake. This may also serve as a counteracting behavior in order to attenuate the effects from frequent disinhibited eating. Thus, as both restraint and disinhibition are associated with increased body weight the restraint eating periods might fail resulting in disinhibited eating episodes which would ultimately lead to higher BMI (Gallant et al. 2010). Further, it has to be mentioned that albeit not significant, we detected higher intake of consumer goods such as alcohol, coffee, and cigarette smoking in homozygous G allele carriers in our discovery cohort.

Our data prompted us to replicate the finding in two other independent cohorts, the German cohort and the Old Order Amish population. We identified in both populations the same effect direction as in the Sorbs but did not reach statistical significance which might most likely be due to low sample size. A weighted meta-analysis of all three cohorts revealed nominal associations of rs2237781 with increased restraint scores implying the variant might be involved in restraint eating to some extent. However, our data need to be interpreted with caution and can be viewed as a suggestive indication that rs2237781 may play a role in influencing restraint scores, especially in our discovery cohort.

Further, in the Old Order Amish we found significant associations with susceptibility to recognizing hunger feelings. It is noteworthy to point out that the three eating behavior factors restraint, disinhibition, and hunger are not considered to be totally independent from each other and thus rs2237781 might be involved in the development of different eating behavior factors influencing individual food intake.

Moreover, it needs to be mentioned that our study is limited at several aspects. First of all, the sample sizes of our study populations are quite small which may have prevented us from significant replication. Second, we cannot rule out that various genetic backgrounds of the studied cohorts, especially the Old Order Amish, may have influenced the heterogeneous outcome of the studies. Third, data regarding consumer goods intake are available for the Sorbs only. Therefore, larger studies are necessary to verify the effects we have detected so far.

It is further noteworthy to acknowledge that, especially in the context of potential functionality, rs2237781 maps near an additional gene encoding microRNA592. One might hypothesize that the SNP may potentially affect posttranslational modifications of GRM8 via regulating the expression of microRNA592. However, further studies are warranted to investigate underlying functional mechanisms.

In conclusion, the present study suggests that rs2237781 within *GRM8* may influence the regulation of human eating behavior and might potentially be involved in affecting human liability to addiction behavior.
